# Transcriptome analysis of upland cotton revealed novel pathways to scavenge reactive oxygen species (ROS) responding to Na_2_SO_4_ tolerance

**DOI:** 10.1038/s41598-021-87999-x

**Published:** 2021-04-21

**Authors:** Qinqin Wang, Xuke Lu, Xiugui Chen, Waqar Afzal Malik, Delong Wang, Lanjie Zhao, Junjuan Wang, Shuai Wang, Lixue Guo, Ruifeng Cui, Mingge Han, Cun Rui, Yuexin Zhang, Yapeng Fan, Chao Chen, Wuwei Ye

**Affiliations:** State Key Laboratory of Cotton Biology, Key Laboratory for Cotton Genetic Improvement, MOA, Institute of Cotton Research of Chinese Academy of Agricultural Sciences, Anyang, 455000 Henan China

**Keywords:** Genetics, Gene expression, Gene regulation

## Abstract

Salinity is an extensive and adverse environmental stress to crop plants across the globe, and a major abiotic constraint responsible for limited crop production threatening the crop security. Soil salinization is a widespread problem across the globe, threatening the crop production and food security. Salinity impairs plant growth and development via reduction in osmotic potential, cytotoxicity due to excessive uptake of ions such as sodium (Na^+^) and chloride (Cl^−^), and nutritional imbalance. Cotton, being the most cultivated crop on saline-alkaline soils, it is of great importance to elucidate the mechanisms involved in Na_2_SO_4_ tolerance which is still lacking in upland cotton. Zhong 9835, a Na_2_SO_4_ resistant cultivar was screened for transcriptomic studies through various levels of Na_2_SO_4_ treatments, which results into identification of 3329 differentially expressed genes (DEGs) in roots, stems and leave at 300 mM Na_2_SO_4_ stress till 12 h in compared to control. According to gene functional annotation analysis, genes involved in reactive oxygen species (ROS) scavenging system including osmotic stress and ion toxicity were significantly up-regulated, especially GST (glutathione transferase). In addition, analysis for sulfur metabolism, results in to identification of two rate limiting enzymes [APR (*Gh_D05G1637*) and OASTL (*Gh_A13G0863*)] during synthesis of GSH from SO_4_^2−^. Furthermore, we also observed a crosstalk of the hormones and TFs (transcription factors) enriched in hormone signal transduction pathway. Genes related to IAA exceeds the rest of hormones followed by ubiquitin related genes which are greater than TFs. The analysis of the expression profiles of diverse tissues under Na_2_SO_4_ stress and identification of relevant key hub genes in a network crosstalk will provide a strong foundation and valuable clues for genetic improvements of cotton in response to various salt stresses.

## Introduction

Soil salinity is a major environmental factor which limits the growth and development of plants, resulting in decrease in crop productivity and quality^1^. The excess accumulation of soluble salts in the soil surface is referred to as salinization, which has an adverse impact on agricultural production, biodiversity and sustainable development and may results into delay of onset, reduce the rate, and increase the dispersion of germination events, leading to reductions in plant growth and finally crop yield^2^. Cotton, being a semi—halophyte with economic value, is considered to be tolerant to salinity stress^3,4^. So, it is of great importance to study the development of cotton under salt stress and explore new salt resistant genetic material. Salt stress adversely affect the plant growth and development and ultimately decreased the yield. High concentration of Na^+^/ Cl^−^ ions in soil are the basic causative agent of salt stress, which breaks the ion and water potential balance in plants, causing phytotoxicity, diminishing plant growth, and ultimately lead to death, resulting limiting crop yield^5^. Because of that, plant cell is subject to high osmotic pressure under high salt condition, resulting in physiological drought, ionic toxicity and malnutrition with a high ion concentration^6^. Saline-alkali stress performed ionic toxicity and water shortage in leaves, which inevitably affected the synthesis of chlorophyll, and then affect the photosynthesis of plants^7^. Therefore, the direct damage of salt stress to plants is mainly reflected in two aspects: ionic toxicity and osmotic stress^8^.

Presence of excessive salts in soil is hazardous for plants normal growth and development due to toxic ions like of Na^+^, K^+^ and Cl^−^ ions. and other ions^9,10^. To cope with salt stress, plants have evolved mechanisms in order to coordinate the activities of various ion transporters and thereby maintain ionic homeostasis in the cell^10^. In Arabidopsis, *SOS* (Salt Overly Sensitive) system related to salt stress was first elucidated significantly in Na^+^ homeostasis^10,11^. Under salt stress, the calcium sensor *SOS*_*3*_ interacts and activates the serine/threonine protein kinase *SOS*_*2*_ with an increase in cytosolic free calcium levels, which results in the activation of the plasma membrane-localized Na^+^/H^+^ antiporter *SOS*_*1*_ to export the Na^+^ out of the cell to prevent the accumulation of toxic material^12^. *SOS*_*1*_ plays a critical role in Na^+^ homeostasis against oxidative stress responses under salt stress in Arabidopsis^11^. *SOS*_*2*_ also positively controls the activities of tonoplast Na^+^/H^+^ antiporter by Sequestering the Na^+^ ions in vacuole and exchange of H^+^/Ca^2+^ ions through *CAX1*^11^.

The Ca^2+^ signaling pathway and ion transport showed the crosstalk with SOS system to enhance salt tolerance^13^. Ca^2+^ binding proteins mainly include *CDPKs* (calcium-dependent protein kinase) containing the catalytic domain of serine/threonine protein kinase, *CAMs* (calcium receptors calmodulin) with an EF-hand unit that binds Ca^2+^, and *CBLs* (Calcineurin B-like proteins) which consist of a single *CIPKs* protein^14^. These have been shown to be involved in salt stress responses, such as *OsCPK21* in rice^15^, *GSCBRLK* in soybean^16^, *TaCIPK25* in wheat^16^, and *SiCBL4* in millet^17^. *CBL9* and *CIPK23* increase salt tolerance at root tips mostly by scavenging reactive oxygen^18^. *CBL10* regulates other unknown transport processes that act as Na^+^ equilibriums different from those regulated by *SOS*_*1*_^19^. *AtGLR3.4* regulates Ca^2+^ flow and controls Na^+^ accumulation through *SOS* pathway during salt-stress seed germination^20^. In addition, the Ca^2+^ signaling pathway also plays a central role in response to ROS scavenging under salt stress and induced programmed cell death through *MAPK* cascade in mitochondria. To cope with the oxidative damage resulting from ROS, not only the enzymatic antioxidants (*SOD, APX, PRX, GPX, CAT, GRX and TRX*) and non-enzymatic scavengers (*ASA*, *GSH*, metabolite of proline, tocopherols, carotenoids and phenolic compounds)^21^, but also the hormones signaling pathway with a lot of transcription factors (TFs), such as abscisic acid (*ABA*) signaling pathway^22,23^, developed a complex regulatory network for plant growth. Previous studies have shown that the majority of plant hormones related to salt stress are melatonin, *ETH* (ethylene), *BR* (brassinolactone), *ASA* (ascorbic acid), *IAA/auxin* and *ABA* (abscisic acid) ^24,25^. These hormones mainly increases the plant salt tolerance by scavenging active oxygen^26^. In recent years, there have been a large number of studies on the relationship between *ABA* and plant salt tolerance. Among them, salt stress can induce the accumulation of endogenous *ABA*, and then induce the expression of *E3* ubiquitin ligase or others transcription factors^25,27^, ion transporter^22^, antioxidant enzyme^28^ and other salt stress-related genes to enhance plant salt tolerance^29^.

For ion transport, *NHX1* regulates the export and import of Na^+^ in and out of vacuoles^10^, and *HKT1* transporters have been found to reduce Na^+^ toxicity by regulating Na^+^/K^+^ balance in several species^30^. In plant cells, the proton pump, including *H*^+^*-PPase* (proton pump pyrophosphoric acid hydrolase), *V-PPase* (vacuole proton pump *ATP* hydrolysis enzyme) and *PMH-ATPase* (plasma membrane proton pump *ATP* hydrolysis enzyme) on the one hand provides energy for cellular metabolism, maintain normal metabolism and cell growth^31^. On the other hand, proton pump such as *H*^+^*-PPases* can improve the salt-resistance^32^. In Arabidopsis, *14-3-3* proteins in the *SOS* system are able to regulate the *V-PPase* (*EC 3.6.1.1*) gene *AVP1* and mitigate the harm of Na^+^^33^. Besides, aquaporin protein, as a transporter of water molecules, responds to salt stress mainly by increasing antioxidant activity and maintaining ion balance^34^. The aquaporin protein related to salt resistance is mainly concentrated on the *PIPs* (Plasma membrane intrinsic proteins) gene. In Arabidopsis thaliana, *Tip2* (Tonoplast intrinsic protein) regulates *MDA* (malondialdehyde) and salt-tolerance related genes (*SOS*_*1*_, *SOS*_*2*_, *SOS*_*3*_, *DREB1A* and *P5CS1*)^35^. *GhSIP* may be involved in endoplasmic reticulum osmotic equilibrium^36^.

Soil salinization has always been a major problem in global agriculture, because of inadequate irrigation and climate change, the area of saline-alkali land is likely to increase with the passage time^37^, so it is of great significance to study the salt tolerance in plants. The ions commonly present in saline soil are Na^+^, Cl^−^ and SO_4_^2−^ ^38^. There are many studies on NaCl, however, few on Na_2_SO_4_. SO_4_^2−^ is different from the Na^+^, K^+^ and Cl^−^^39^, and Na_2_SO_4_ is more toxic than NaCl^40,41^. *Prosopis Strombulifera* (Lam.) Benth, a kind of halophytic shrub with high NaCl tolerance, is found in high saline-alkali soil in *Argentina*, but affected with decreased Fv/Fm, ETR and Y(II) photosynthetic parameters significantly under Na_2_SO_4_ condition^40^. While, in *Kalidium foliatum* the activity of Rubisco (Ribulose-1,5-bisphosphate Carboxylase/Oxygenase) treated with NaCl was higher than that of treated with 100 mM Na_2_SO_4_, and there was no significant change under NaCl + Na_2_SO_4_ mixture treatment^42^. In *Brassica rapa*, the result of gene analysis of *GSLs* (Glucosinolates) biosynthetic pathway and transcription factor showed that sulfate had the strongest inhibition on growth under different treatment with NaCl, Na_2_SO_4_, KCl and K_2_SO_4_, respectively^41^.

So far, there is a lack of research on Na_2_SO_4_ salt stress, especially in cotton, and the mechanism of its toxicity is still unclear. In this study, through the analysis of the phenotypic, physiological and biochemical indexes and morphological analysis of Zhong 9835 in response to salt stress during 0 h, 6 h, 12 h and 24 h, we found that it is best to analysis Zhong 9835 transcriptome at 12 h under 300 mM Na_2_SO_4_ treatment. Sulfur metabolism was enriched in 3329 differentially expressed genes (DEGs) among roots, stems and leaves, especially GST, followed by Ubiquitin transcription factors. This study not only provides complement data for regulatory network at early stage under Na_2_SO_4_ stress but also provides a strong foundation and valuable clues for genetic improvements of cotton in response to various salt stresses.

## Results

### Phenotypic responses of Zhong 9835 to Na_2_SO_4_ stress

**Figure 1:**
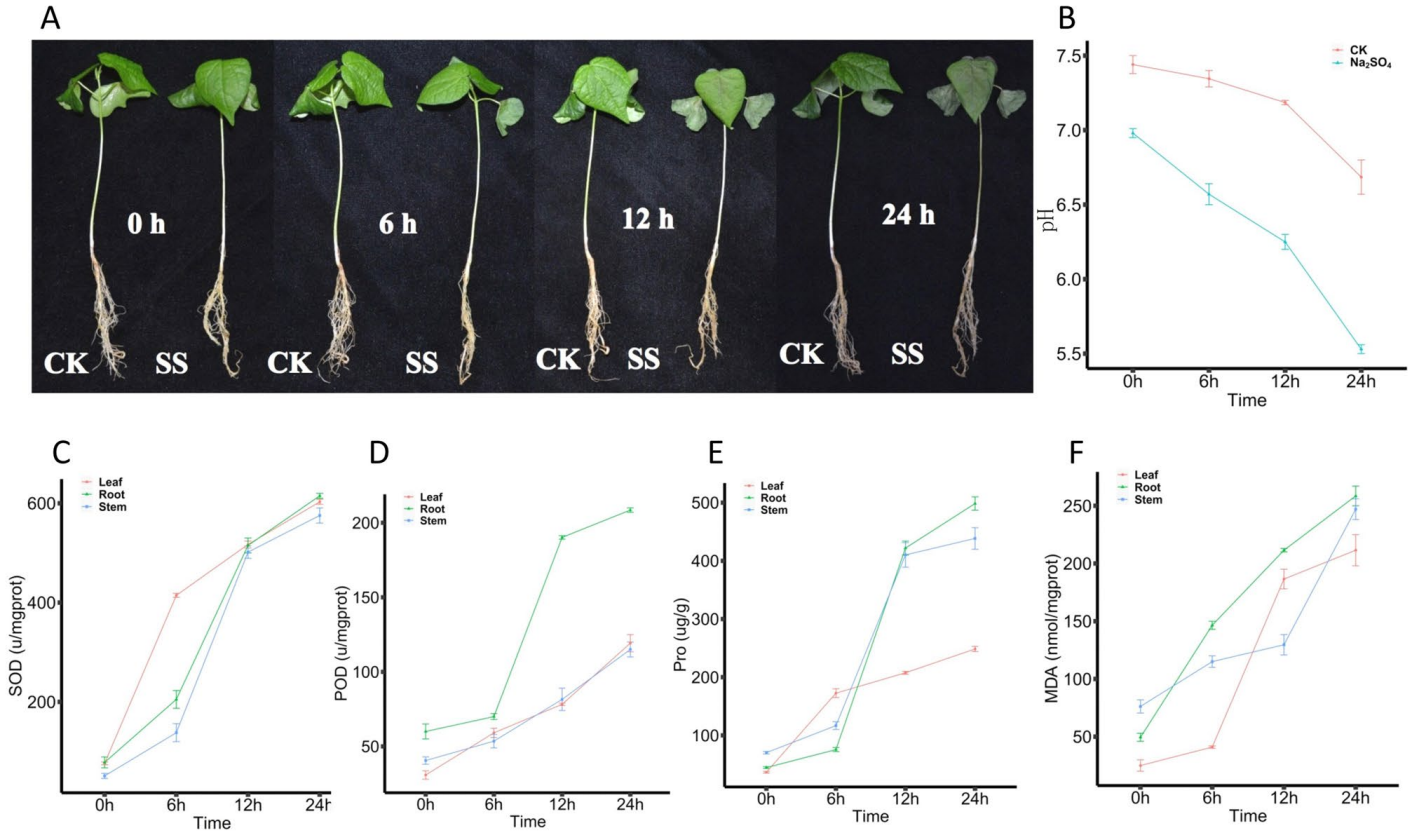
Phenotypic, physiological and biochemical indexes and morphological analysis of Zhong 9835 in response to salt stress. CK: Control group; SS: 300 mM Na_2_SO_4_. (**A**) Phenotypic changes of Zhong 9835 under the SS and CK during 0 h, 6 h, 12 h and 24 h. (**B**) pH value changes of water under the SS and CK during 0 h, 6 h, 12 h and 24 h. (**C**) The level changes of SOD (Superoxide dismutase) under the SS and CK during 0 h, 6 h, 12 h and 24 h. (**D**) The level changes of Peroxidase (POD) under the SS and CK during 0 h, 6 h, 12 h and 24 h. (**E**) The changes of Proline (Pro) Content under the SS and CK during 0 h, 6 h, 12 h and 24 h. (**F**) The changes of Malondialdehyde (MDA) content under the SS and CK during 0 h, 6 h, 12 h and 24 h.

Previous studies have reported that cotton is more sensitive to abiotic stresses at three-leaf stage^43^. Different morphological data has been observed in *G. hirsutum* Zhong 9835 during its three-leaf stage under various concentrations of Na_2_SO_4_ stress. We observed the significant phenotypic difference among roots, leaves and shoots of cotton seedlings under 300 mM Na_2_SO_4_ stress after 12 h of treatment as compared to control conditions without any salt treatment (Fig. 1A). After 12 h of treatment, leaves start wilting, and roots gradually truing into black (Fig. 1A), while after 24 h, leaves and stems were seriously wilted (Fig. 1A). The result showed that under 300 mM Na_2_SO_4_ solution the pH value ranges from 6.93 at 0 h to 5.6 at 24 h, among which it becomes more acidic after 6 h (Fig. 1B). The minimum pH value was noticed at 12 h to 24 h under the treatment of Na_2_SO_4_ and control, especially in control from 7 to 6.5 (Fig. 1B). Meanwhile, SOD, POD, Proline and MDA contents in roots, stems and leaves all increased gradually from 0 to 24 h (Fig. 1 C/D/E/F). Among which, SOD up to a same content among root, stem and leaf, especially largest increase from 6 to 12 h in root and stem (Fig. 1C). POD of root increased widely from 6 to 12 h (Fig. 1D). Substantial increase of proline content of root and stem was observed at 6 h to 12 h (Fig. 1E), MDA of leaf increased greatly from 6 to 12 h, while root amplitude increased from 0 to 6 h and stem from 12 to 24 h (Fig. 1F).

### Transcriptome sequencing and alignment

Two groups (treated versus control) with three biological replications were conducted among the samples of roots, stems, and leaves, respectively. Totally 18 qualified libraries were established from the tissues of the roots, stems and leaves at 12 h with salt stress and control conditions. On average, of 42.5 million raw reads for the 18 libraries were obtained by using an Illumina Novaseq 6000 sequencing platform (Table 1). 111.34 Gb (Gigabyte) of sequence data and over 96% of the clean reads with a Q30 level were done through approximately 742 million clean valid reads. With the process of adaptor deletion, junk filtering and low copy filtering, > 95% of the sequences were confirmed as clean data, which then mapped to cotton whole genome (*G. hirsutum*) by using His at software^44^. In final, > 93.94% of the total reads were mapped to the reference genome, while the unique mapped reads were 63.46%-70.27% by the String Tie software^45^.

**Table 1:** Summary of the DGE sequencing tags and their matches in the *G. hirsutum* genome.

Sample	Raw reads (M)	Clean reads (M)	GC content	≥ Q30	Mapped reads (M)	Unique mapped reads (M)	Multi mapped reads (M)
CK_L1	42.2	40.5	44%	97.24%	95.66%	67.57%	28.09%
CK_L2	39.3	37.7	44.5%	97.2%	95.33%	67.42%	27.90%
CK_L3	38.1	37.1	45%	96.87%	94.36%	66.19%	28.18%
CK_R1	50.8	49.6	43%	97.13%	94.02%	67.61%	26.41%
CK_R2	35.8	34.6	43%	97.14%	93.78%	68.15%	25.64%
CK_R3	46.7	45.4	43%	97.23%	95.16%	69.63%	25.53%
CK_S1	39.1	37.6	43%	97.43%	94.70%	69.23%	25.48%
CK_S2	34.7	33.5	43%	97.02%	93.69%	68.39%	25.29%
CK_S3	42.4	40.7	43%	97.18%	93.93%	68.30%	25.63%
SS_L1	41.2	40.1	44%	97.19%	95.88%	69.68%	26.20%
SS_L2	42.3	41.1	43.5%	96.83%	95.48%	69.22%	26.26%
SS_L3	41.9	40.9	44%	97.42%	95.88%	69.26%	26.62%
SS_R1	40.4	39.3	44%	97.08%	90.85%	67.10%	23.75%
SS_R2	42.9	41.4	44.5%	97.4%	87.03%	63.46%	23.58%
SS_R3	49.9	47.7	44%	97.48%	90.92%	66.12%	24.80%
SS_S1	55.4	54.0	43%	96.99%	93.27%	67.66%	25.61%
SS_S2	44.1	43.2	43%	97.16%	95.73%	70.01%	25.72%
SS_S3	38.4	37.6	43%	97.06%	95.24%	70.27%	24.98%

### Exploration of DEGs in roots, stems and leaves in response to Na_2_SO_4_ stress

**Figure 2:**
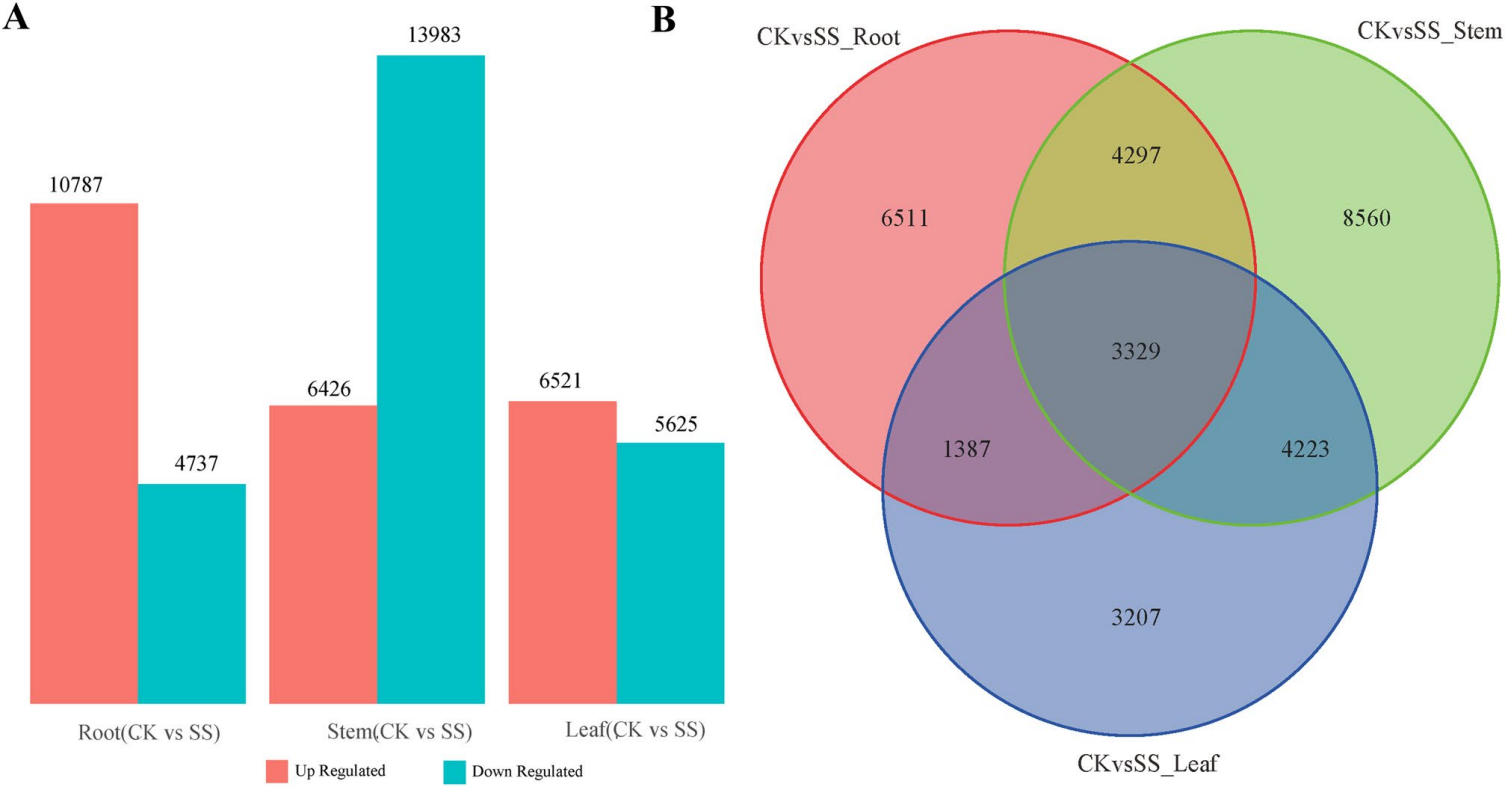
Expression dynamics changes and comparative analysis of differentially-expressed genes (DEGs) among CK_R, SS_R, CK_S, SS_S, CK_L, SS_L. CK: Control group; SS: 300 mM Na_2_SO_4_. **(A)** Number of up-regulated and down-regulated DEGs of each sample between SS and CK. **(B)** Number of DEGs among roots, stems and leaves under SS and CK treatments.

Gene expression levels were estimated by fragments per kilo base of transcript per million fragments mapped (FPKM). Differential expression analysis of treatments and control group was performed using the DESeq among CK_R, SS_R CK_S, SS_S, CK_L, SS_L. According to root, stem, and leaf, transcriptome data between control and treatment with Na_2_SO_4_ were divided into 3 groups (CK_R vs SS_R, CK_S vs SS_S, CK_L vs SS_L) (Fig. 2A,B). The samples from Root (CK_R vs SS_R) showed 15,524 DEGs, among which 10,787 genes were up-regulated and 4,737 genes were down-regulated (Fig. 2A). A total of 20,409 genes were differentially expressed in the samples of Stem (CK_S vs SS_S), among which 6,426 genes were up-regulated and 13,983 genes were down-regulated (Fig. 2A). There were 12,146 DEGs identified from the Leaf sample (CK_L vs SS_L), among which 6,521 genes were up-regulated and 5,625 genes were down-regulated (Fig. 2A).

To further explore the DEGs between different groups, we sorted the common genes using a Venn diagram online tool (https://bioinfogp.cnb.csic.es/tools/venny/). 7,626 DEGs were identified between the samples CK_R vs SS_R and CK _S vs SS_S (Fig. 2B). among the samples of CK_R vs SS_R and CK _L vs SS_L, there were 4,716 DEGs (Fig. 2B). A total of 7,552 DEGs were identified from the samples CK_S vs SS_S and CK_L vs SS_L (Fig. 2B). finally, we found 3,329 DEGs among the samples of CK_R vs SS_R, CK_S vs SS_S and CK _L vs SS_L (Fig. 2B).

### Validation of RNA-Seq data by quantitative real-time PCR

**Figure 3:**
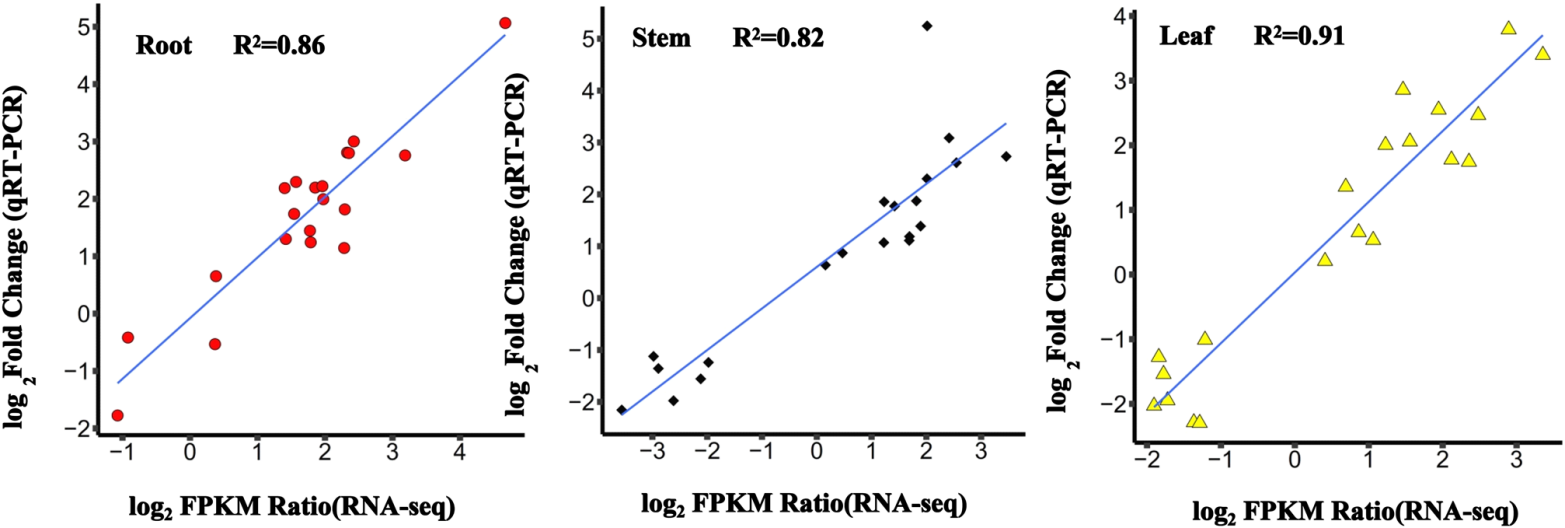
qRT-PCR validation of transcript levels evaluated by RNA-Seq in roots, stems and leaves under 300 mM Na_2_SO_4_ stress conditions. X-axis represents log_2_FC (Fold Change) derived from RNA-seq; Y-axis represents log_2_(2^−ΔΔCt^) specifically from the qRT-PCR experiment. **(A)** Transcript level of roots. **(B)** Transcript level of stems. **(C)** Transcript level of leaves.

In order to validate the differential expression analysis of RNA-seq data, we performed the quantitative real-time PCR (qRT-PCR) of 20 genes to confirm the reliability of RNA-seq data by using the same RNA samples (Table S1). To corroborate the expression levels measured by RNA-Seq data, the ratio of expression levels among root, stem and leaf under Na_2_SO_4_ stress and control using RNA-Seq was compared to the ratios of expression measured by qRT-PCR. The results showed that there was a good correlation between RNA-Seq and real-time PCR results among three tissues (coefficient of determination R^2^ = 0.86, 0.82 and 0.91) (Fig. 3A–C). The validation experiments support the accuracy of the RNA-Seq quantification of gene expression by relative values provided by the qRT-PCR analysis.

**Figure 4:**
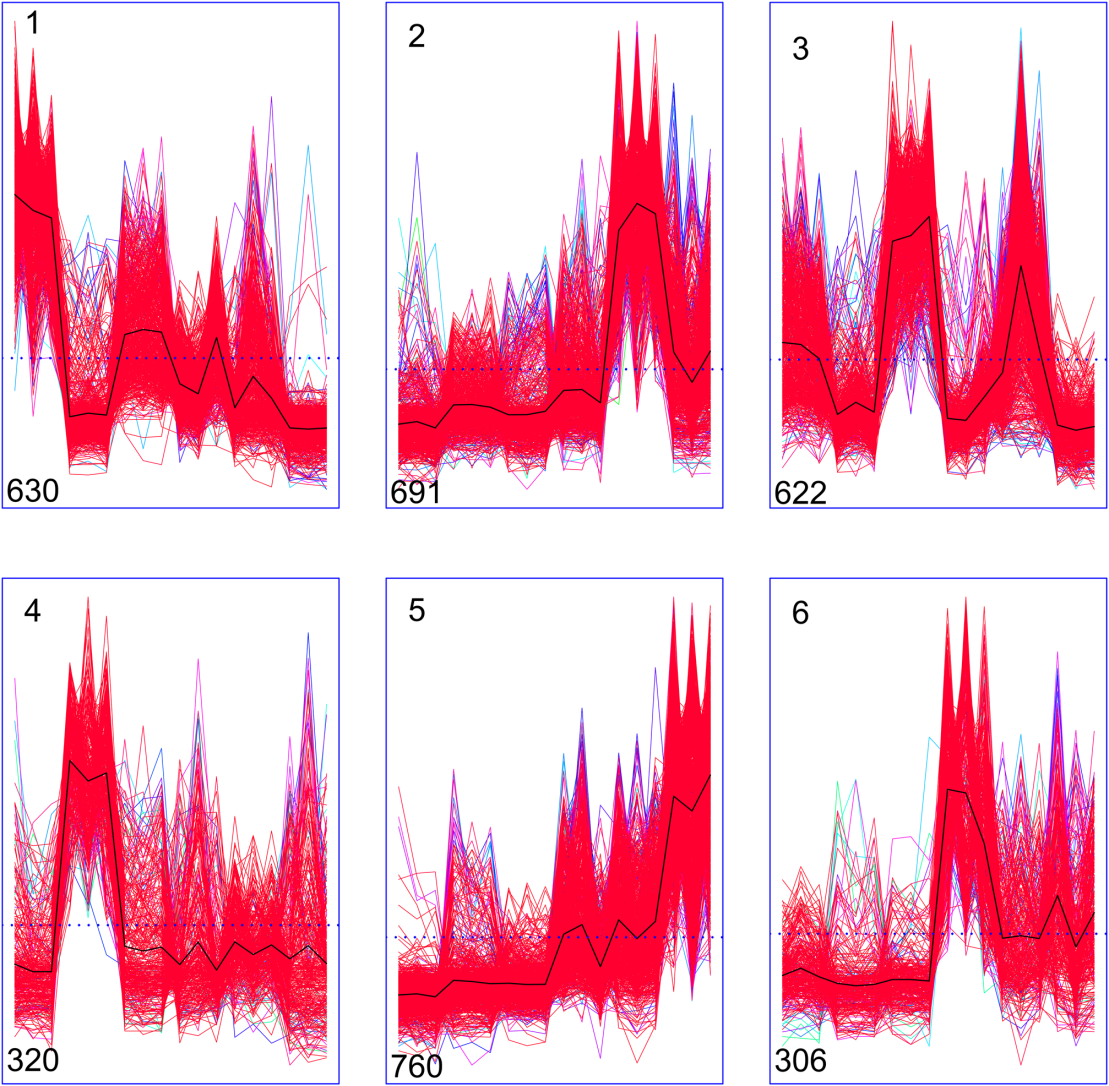
Line graph for the cluster expression of 3329 DEGs. CK: Control group; SS: 300 mM Na_2_SO_4_. The X-axis shows the different treatments (from left to right: CK_L1, CK_L2, CK_L3, CK_R1, CK_R2, CK_R3, CK_S1, CK_S2, CK_S3, SS_L1, SS_L2, SS_L3, SS_R1, SS_R2, SS_R3, SS_S1, SS_S2, SS_S3), and the Y-axis shows the standardized FPKM. The dotted line shows the 0 value of FPKM. The number on the top left side of cluster panel is cluster number. The number on the bottom left side of cluster panel is genes number of each cluster. Black lines represent the average value of the relative expression level of all genes included in the cluster.

To study the clustering profiles of these 3329 DEGs, six samples with three biological repeats were carried out (Fig. 4). These 3329 DEGs were divided into six clusters, in which cluster had the same expression profile (Fig. 4). There were 630 genes in cluster 1, which had higher FPKM values especially in CK_L group with three biological repeats. In cluster 2, the samples SS_R possess 691 genes with three replication and harbor higher FPKM values. The FPKM values of cluster 3 were found higher than others in samples CK_S with three biological repeats, which includes 622 genes. Cluster 4 containing 320 genes was the smaller one in the clustering profile, in which the FPKM is greater in samples CK_R with three biological repeats particularly. Cluster 5 with 760 genes was the largest one in the clustering profile, which the FPKM of the samples SS_S with three biological repeats is higher. Cluster 6 of 306 genes was the smallest one, in which the FPKM of the samples SS_L with three biological repeats is greater.

### Functional enrichment of differentially-expressed genes

**Figure 5:**
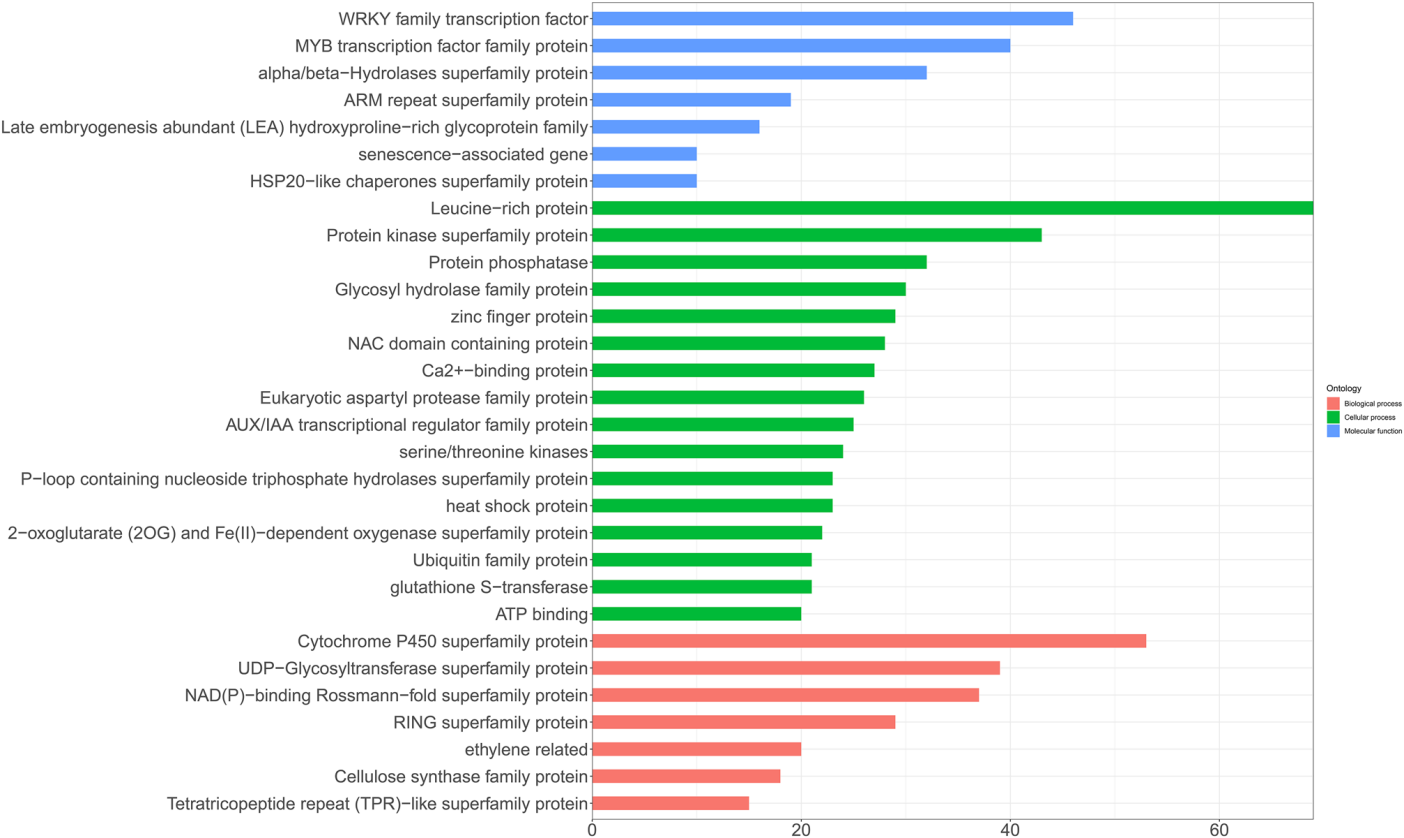
Gene ontology functional classification of DEGs drawn by ggplot2 (v3.3.3). X-axis represents the number of genes; Y-axis represents the GO terms names: red pillars represent biological process, green pillars represent cellular process, and blue pillars represent molecular function.

**Figure 6:**
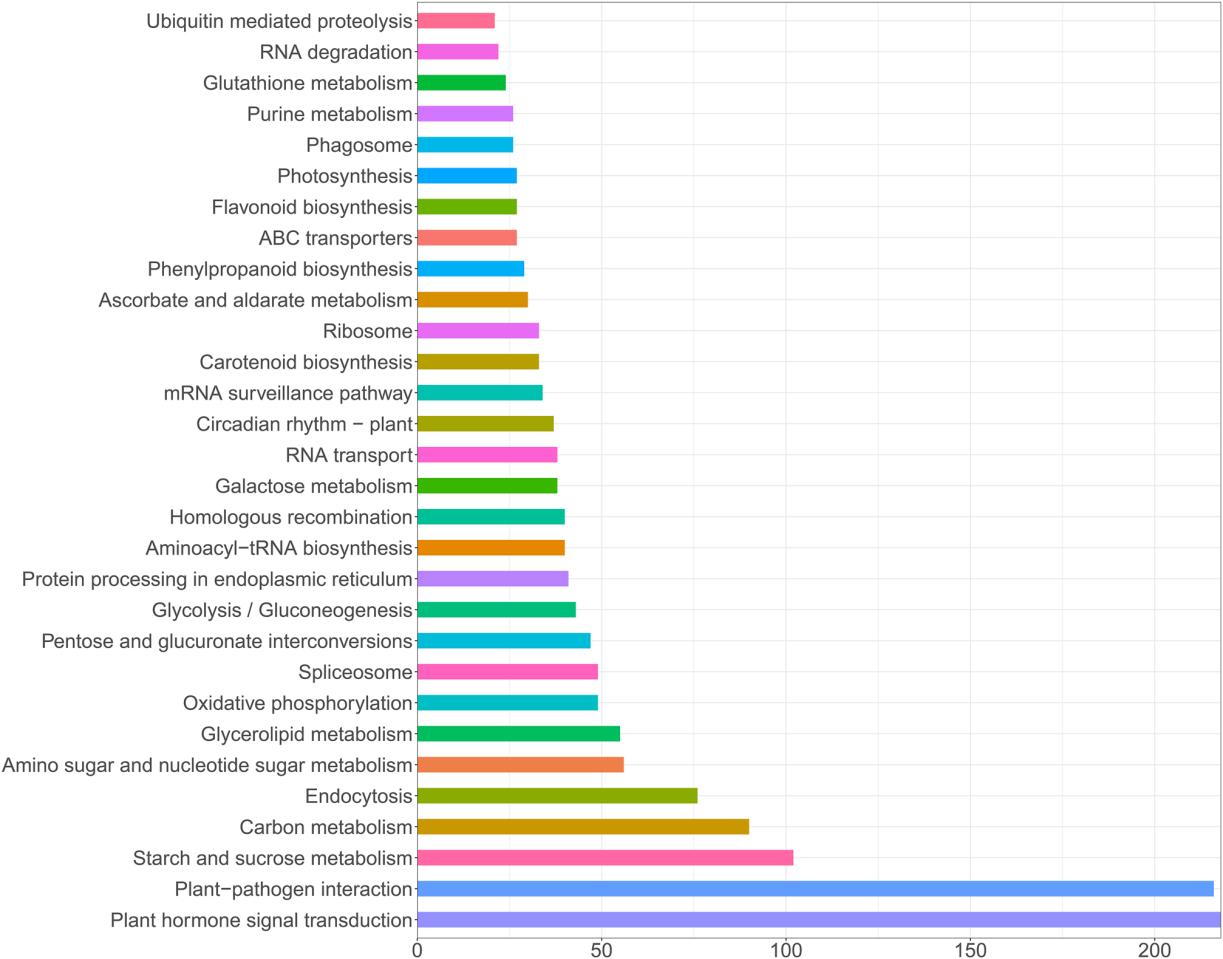
KEGG pathway enrichment analysis of DEGs drawn by ggplot2 (v3.3.3). X-axis represents the number of genes; Y-axis represents the name of KEGG pathway: the different colour represents different KEGG pathway.

To further understand the molecular mechanism of 3329 DEGs, we mapped all of DEGs to the GO database and the Kyoto Encyclopedia of Genes and Genomes (KEGG) pathways^46^. In final, the identified 3,329 DEGs were classified into 100 pathways (1877 DEGs, account for 56.90%) and 383 Gene Ontology (GO) annotations (3144 DEGs, accounts for 95.30%) which includes the biological processes, cellular components and molecular functions. Then the first 30 GO terms (Fig. 5) and a threshold of top 30 set for KEGG pathways analysis (Fig. 6) were chosen.

**Figure 7:**
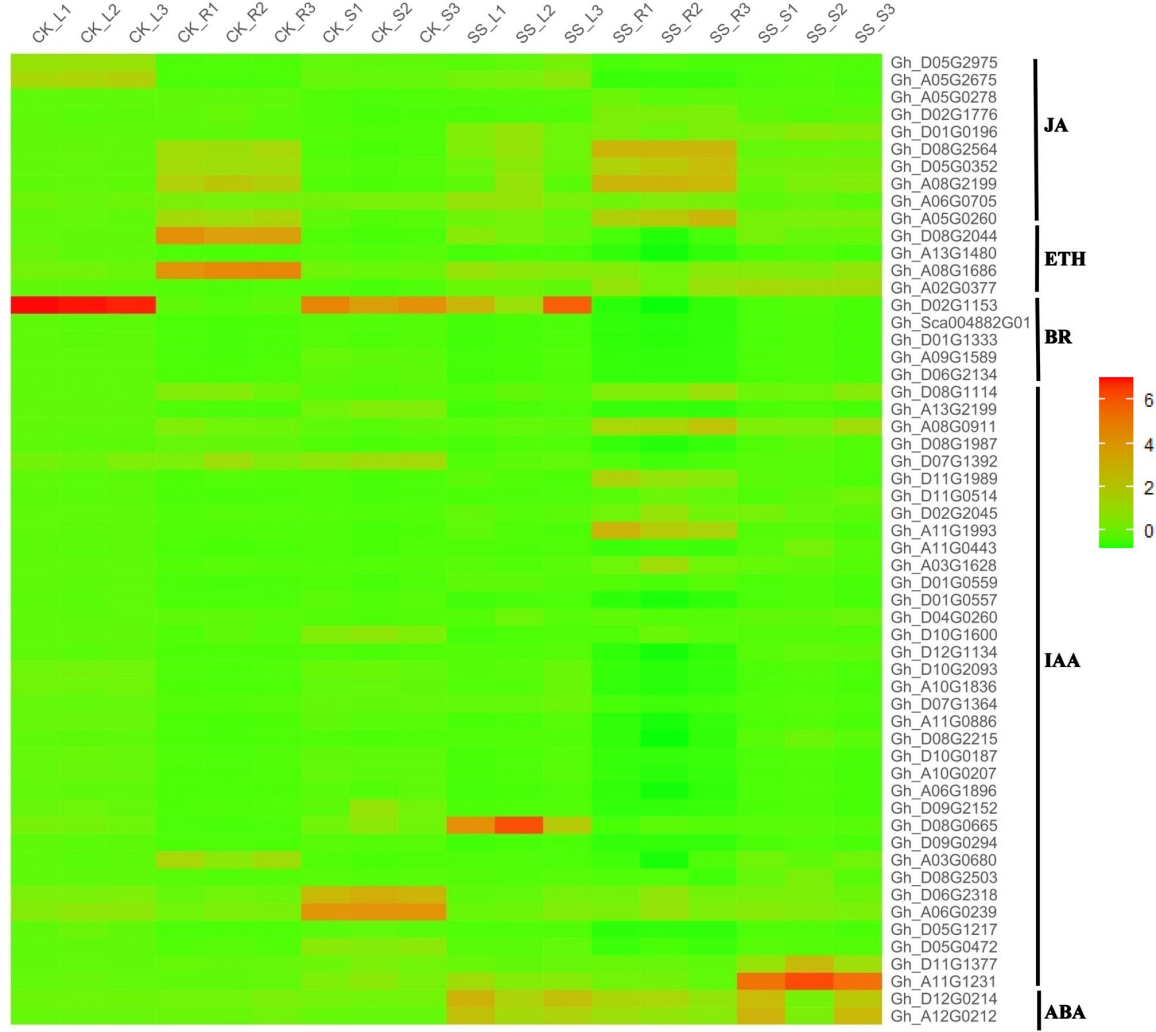
Heatmap of the standardized FPKM expression level of the DEGs related to hormones enrichment among roots, stems and leaves between SS and CK drawn by ggplot2 (v3.3.3). CK: Control group; SS: 300 mM Na_2_SO_4_. Red = high expression level of genes, and Green = low expression level of genes.

**Figure 8:**
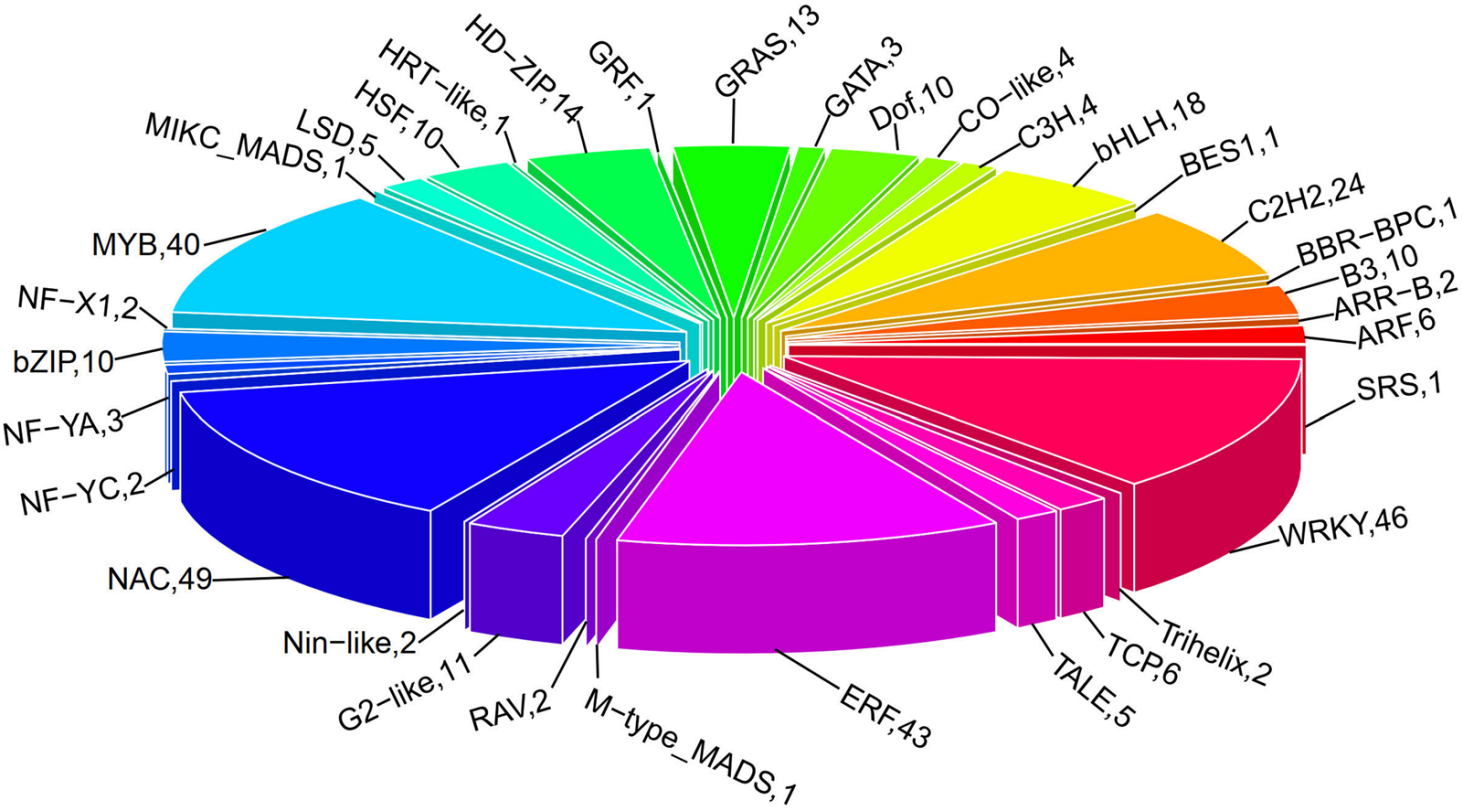
Annotation of transcription factors of specific DEGs under Na_2_SO_4_ stress created by plotrix (v3.8.1).

**Figure 9:**
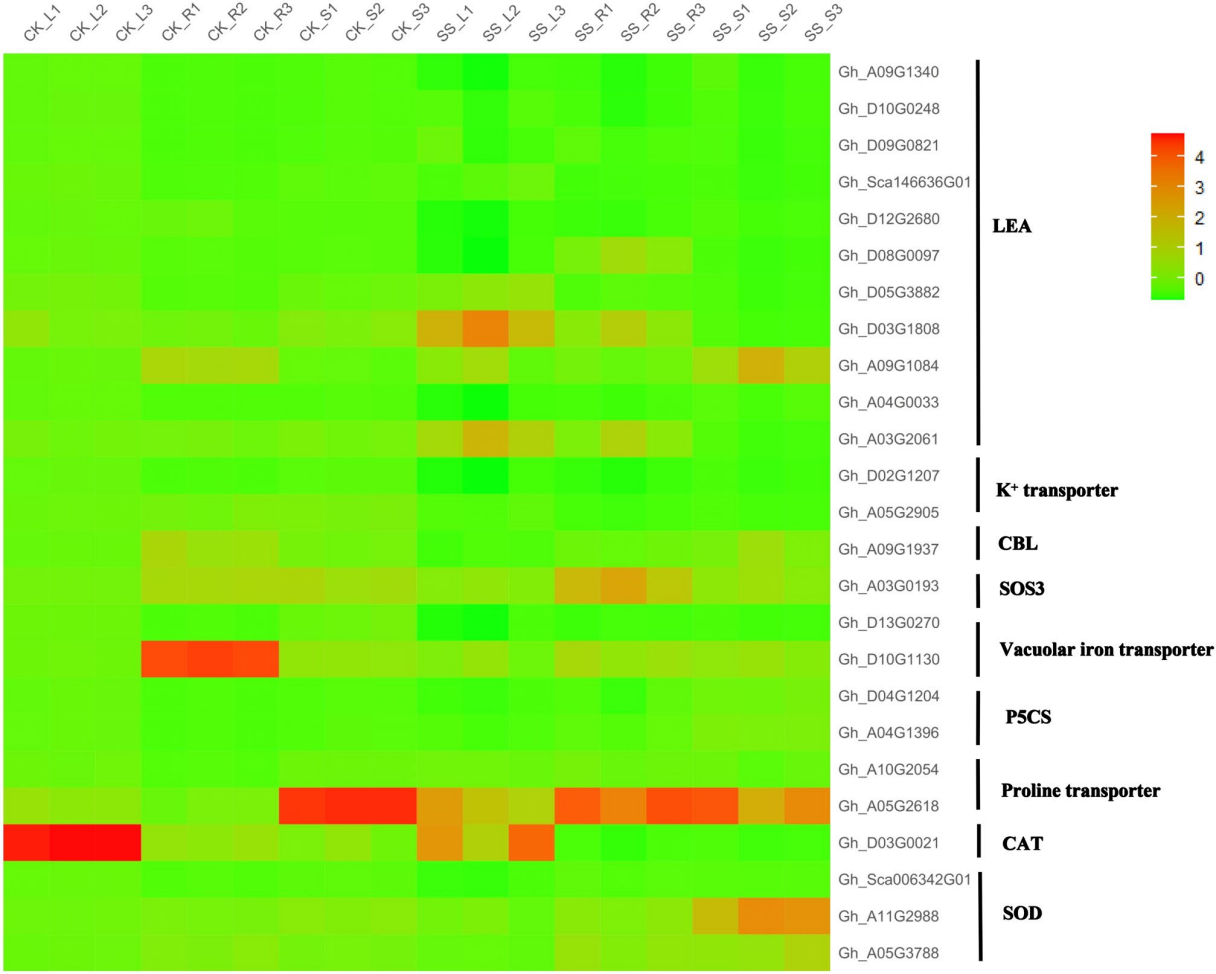
Heatmap of the standardized FPKM expression level of the DEGs related to ionic homeostasis enrichment among roots, stems and leaves between SS and CK performed by ggplot2 (v3.3.3). CK: Control group; SS: 300 mM Na_2_SO_4_. Red = high expression level of genes, and Green = low expression level of genes.

“Molecular function” (GO:0003674) was enriched, which mainly contain WRKY family transcription factor (46 DEGs), MYB transcription factor family protein (40 DEGs), Late embryogenesis abundant (LEA) hydroxyproline-rich glycoprotein family (16 DEGs) and *HSP20-like* chaperones superfamily protein (10 DEGs). In biological process, these genes were mostly Cytochrome P450 superfamily protein (53 DEGs), UDP-Glycosyltransferase superfamily protein (39 DEGs), NAD(P)-binding Rossman-fold superfamily protein (37 DEGs), RING superfamily protein (29 DEGs) and ethylene related (20 DEGs). In cellular process, the maximum number of 136 genes were found in protein kinase (including 69 DEGs of Leucine-rich protein, 43 DEGs of Protein kinase superfamily protein and 24 DEGs of serine/threonine kinases) and Protein phosphatase (32 DEGs), followed by Ca^2+^-binding protein (27 DEGs), heat shock protein (23 DEGs), Ubiquitin family protein (21 DEGs), and ATP binding (20 DEGs). DEGs were significantly enriched in the pathway “04075 (Plant hormone signal transduction)” (218 DEGs, account for 6.61%), “04626 (Plant-pathogen interaction) “(216 DEGs, account for 6.55%), “00500 (Starch and sucrose metabolism)” (102 DEGs, account for 3.09%), “01200 (Carbon metabolism)” (90 DEGs, account for 2.73%). JA (jasmonic acid), ETH (ethylene), BR (brassinosteroids), IAA/auxin and ABA (abscisic acid) were found among 3,329 DEGs (Fig. 7), of which *Gh_A12G0212* and *Gh_D12G0214* associated with ABA hormone signal transduction were up-regulated under Na_2_SO_4_. It is reported that transcription factors play an important role in hormone signaling pathways in response to salt stress^22,47^. 353 transcription factors (TFs) (10.70% among 3,299 DEGs) were up-regulated (Fig. 8), of which NAC (13.88%) was the maximum number of 49 genes, follow by 46 (13.03%) in WRKY, 43 (12.18%) in ERF, 40 (11.33%) in MYB. Besides, there were 108 ubiquitin, accounting for 3.30% of the 3299 DEGs (Figure S6). In addition, ion absorption, compartmentalization and the osmotic balance responded to salt stress, were detected from 3,329 DEGs (Fig. 9), which includes K^+^ transporter, *CBL*, *SOS*_*3*_, iron transporter and SOD, CAT, Proline transporter, *P5CS*, *LEA*^48,49^.

### Metabolism analysis

**Figure 10:**
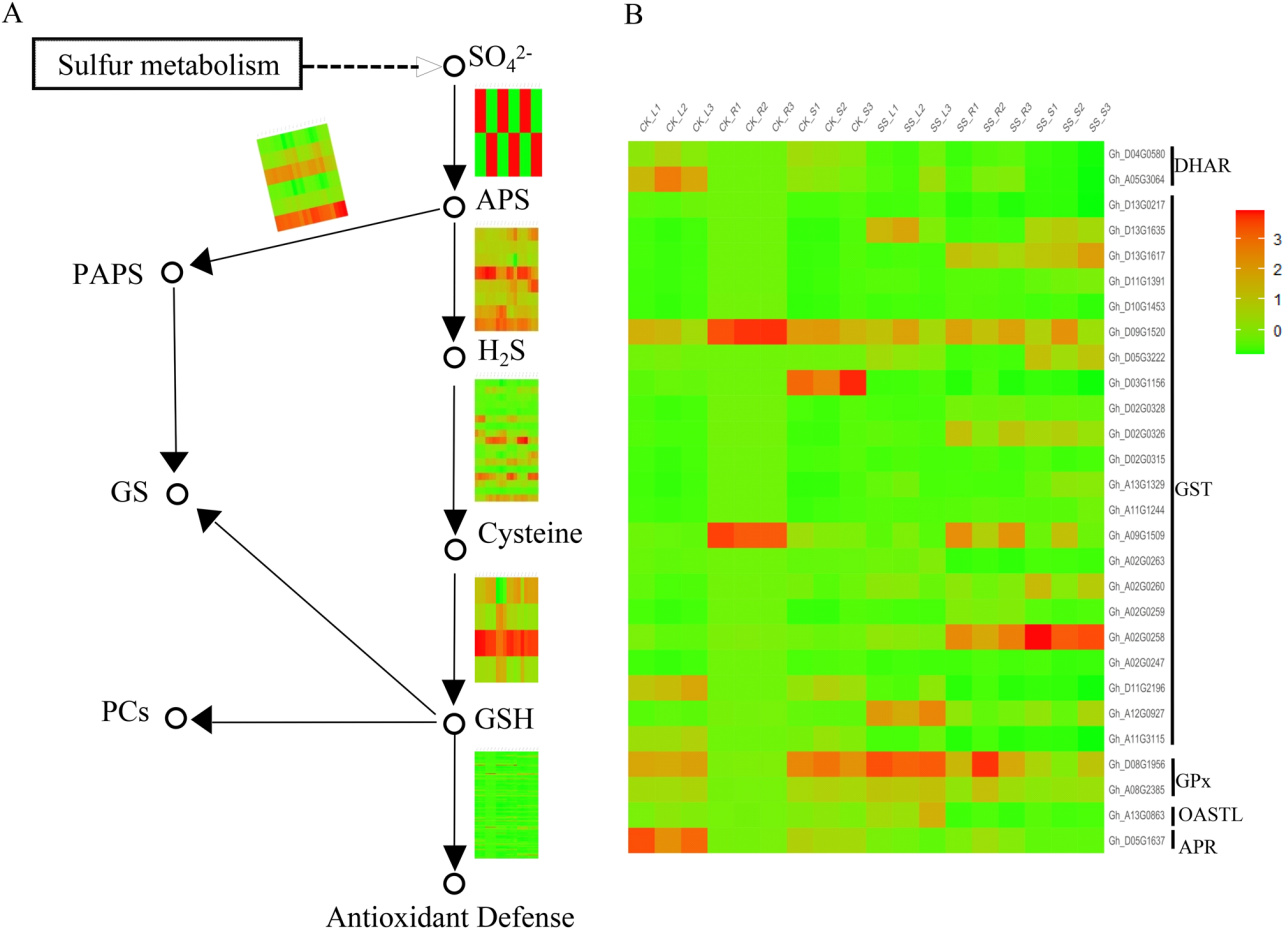
Analysis of differential genes in pathways of sulfur metabolism. CK: Control group; SS: 300 mM Na_2_SO_4_. **(A)** A schematic diagram of sulfur metabolism. Heatmap represents the expression level of regulatory enzyme gene for specific process performed by ggplot2 (v3.3.3). **(B)** Up-regulated DEGs and down-regulated DEGs under CK and SS performed by ggplot2 (v3.3.3).

So far, the research of salt stress about sulfuric acid in the cotton was still lacking. In this study, it is interesting that *GSH*-*ASA* system (Fig. 10A,B), as the roles of antioxidant genes, were significantly up-regulated under Na_2_SO_4_ conditions (Fig. 10B). *GSH-ASA* is an important antioxidant system, of which the performance of *GSH*, as three roles that one for *GS* (glucosinolate) as a storage material of SO_4_^2−^, one for *PCs* (Phytochelatins) as metal-binding oligopeptides in the heavy-metal detoxify mechanism and one for antioxidant system, is most important (Fig. 10A). *APR* (*Gh_D05G1637*) and *OASTL* (*Gh_A13G0863*) identified among 3,299 DEGs are two important rate-limiting enzymes in synthesis of GSH from SO_4_^2−^.

## Discussion

Na_2_SO_4_, being a neutral salt, is made up of Na^+^ and SO_4_^2−^. High concentrations of Na^+^ and SO_4_^2−^ in the soil not only causes the salt toxicity to plants^50^, but also hindered the uptake of other minerals^51^. In our present study, we used Na_2_SO_4_ solution of 300 mM as a salt stress to Zhong 9835 at three leaf stage, in which the pH value ranges from 6.93 at 0 h to 6.2 at 12 h (Fig. 1B). Under the mentioned treatment plants shows significant phenotypic differences among tissues of roots, shoots and leaves. As the roots get start blacking cotyledon turn wilted and leaves lost the turgidity due to loss of water and becomes weak and thin along with stem browning. (Fig. 1A). The pH value at 24 h was 5.6 (Fig. 1B), the root blackened more significantly, the stem browned, the cotyledon shriveled, and the true leaves are wilting and turning brown (Fig. 1A). Previous studies report that the main site of Na^+^ toxicity for most plants is the leaf blade, where Na^+^ accumulates after being deposited in the transpiration stream, rather than in the roots^52^. And an important oxidative damage only to be induced by SO_4_^2−^ anion with an increase in H_2_O_2_ and MDA content^40^, although sulfur could be taken up by the roots and stored in the vacuoles of root and xylem parenchymal cells^53^. In other words, at 12 h root blackening is most likely the toxic phenomenon caused by the oxidation of SO_4_^2−^ in weak acidic solution.

**Figure 11:**
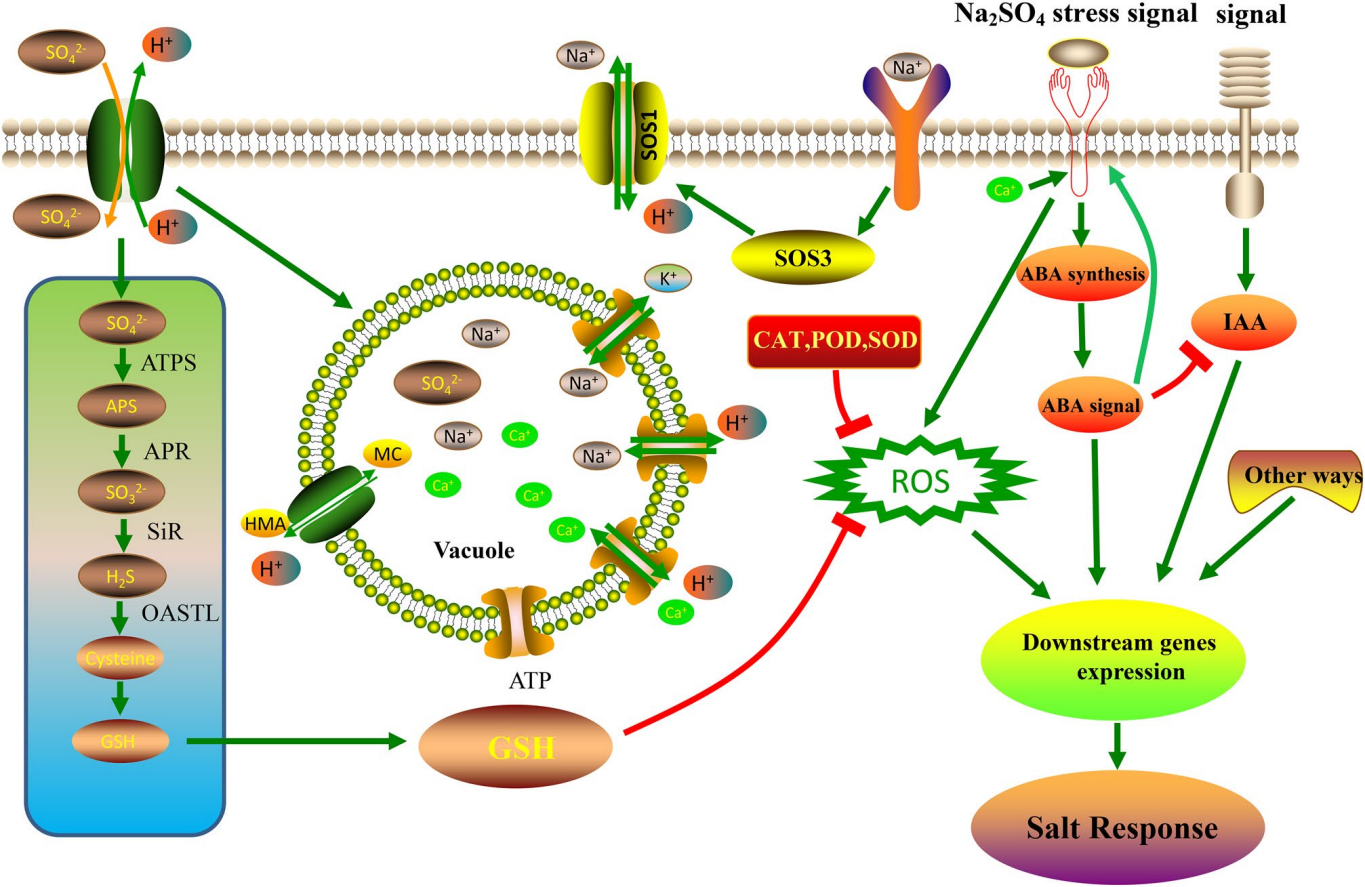
Model of the regulatory networks in response to Na^+^ stress and SO_4_^2−^. The leftmost part of the networks is related to SO_4_^2−^ stress. The others part of the networks is related to ROS, which mainly include Na^+^ stress and ABA signal. The vacuole is the crosstalk of SO_4_^2−^ stress and Na^+^ stress.

Osmotic stress and ionic toxicity can cause oxidative damage^54^. In response to osmotic stress (Figure S3), we found that starch and sucrose metabolism enriched (Fig. 6) and some organic material such as *LEA* (Fig. 9), *HSFs* (Fig. 8), proline and its biosynthesis key enzymes *P5CS* (Fig. 9) is up-regulated consistent with previous research report^49^. As ion transport for a Na^+^ detoxification way (Figure S5), SOS system, on the one hand, can potentially ejected Na^+^ by Na^+^/H^+^ exchangers located in the plasma membrane: on the other hand, sequestered it into the vacuole by Na^+^/H^+^ exchangers (e.g. *NHX* proteins) located in the tonoplast^55^. As previous studies, *HKT1*, as a Na^+^/K^+^ transporters, regulated the equilibrium of the Na^+^/K^+^ decreasing of Na^+^ toxicity^43^. Arabidopsis K^+^ transporter1 (*AKT1*) activity is repressed by *SCaBP8* (CALCINEURIN B-LIKE10 or CBL10, known as SOS3-LIKE CALCIUM-BINDING PROTEIN8, SCaBP8), which interacted with and activated by *SOS*_*3*_-*SOS*_*2*_ complex (Fig. 11) ^56^.

ROS scavenging system includes the enzymatic antioxidants (SOD, APX, PRX, GPX, CAT, GRX and TRX) and non-enzymatic scavengers (GSH-ASA system). It is well known that in cotton the enzymatic antioxidants and non-enzymatic scavengers participated in salt stress^57^. Among root, stem and leaf, not only the enzymatic antioxidants POD and CAT (Fig. 9), but also the non-enzymatic scavengers GPX, GST, DHAR and GST (Fig. 10B) genes were found to be up-expressed under Na_2_SO_4_ condition. And it is interesting that the number of the non-enzymatic scavengers, as a role in the reaction of peroxide detoxification by catalyzing GSH to GSSG^58^, especially GST is more than the enzymatic antioxidants (Fig. 10B). These results suggest that GST may be the major scavengers of ROS under Na_2_SO_4_ stress.

In response to ROS (Figure S1), both the ROS scavenging system and others signaling pathways regulated by transcription factors (Figure S4) work synergistically with efforts in avoiding PCD (Figure S2)^59^. Hormones including JA, ETH, BR, IAA and in particular ABA (Figure S4) although just one gene with two sub-genomes (Fig. 7), under salt stress a mass of researches had reported the crosstalk of ABA and some TFs belonging to others class such as Ubiquitin, MYB, NAC, bZIP and AP2/ERF^60^. Among these transcription factors, ubiquitin is most than others (Fig. 8). In apple, *MdbHLH3* gene (an anthocyanin-related basic helix–loop–helix transcription factor (*bHLH TF*) gene) promotes ethylene production involved in ethylene biosynthesis including *MdACO1*, *MdACS1*, *MdACS5A MdACS1*, and *MdACS5A*^61^. *U-box E3* ubiquitin ligase gene *MdPUB29*, highly homologous with *AtPUB29*, direct ubiquitination of the *MdbHLH3* protein, positively regulating salt tolerance^54^. In addition, *VTC1-CSN5B* associated with the *COP9* signalosome complex promotes ubiquitination-dependent *VTC1* degradation through the 26S proteasome pathway, affecting the response to salt stress by regulating *ASA* synthesis^62^. In hormones, *ETH* and *ABA* induced cell senesce or cell death^62,63^, while auxin, *BR* and *JA* as roles related to abiotic stress can prevent the accumulation of *ROS* from that^64^. For example, IAA/auxin can reverse the hypersensitive response stimulated by purified harpin protein to extent^65^. AUX/IAA-mediated auxin signaling contributes to ethylene-dependent inducible aerenchyma formation in rice roots^66^. To *Arabidopsis* cell suspension cultures, auxin had an effect on the control of cell wall composition and rigidity, preventing the cell death^67^. In present study, the largest number of hormones is *IAA/auxin*, followed by JA and BR (Fig. 7). These results indicate that in Zhong 9835 the root, stem, leaf cell of treatment of 12 h under Na_2_SO_4_ stress is becoming balance by removing ROS toxicity (Fig. 11).

## Materials and methods

### Plant materials and salt stress treatments

The experimental materials in this study “Zhong 9835”, cotton cultivar (*Gossypium hirsutum* L.), was provided by Institute of Cotton Research of Chinese Academy of Agricultural Sciences. The experimental methods were as follows: Seeds were sown in sand soil pots. The sand was washed cleanly and sterilized at 121 °C for 8 h. Sterilized seeds with 1% sodium hypochlorite for 15 min and washed with sterile water for 3 times. The sterilized seeds were grown on sand with a water content of 17% under the condition of 28/25 ℃ long sunshine for 16 h/8 h with a light intensity of 150 μmol·m^−2^·s^−1^. At three true leaf stage, the samples from roots, stems and leaves with three biological repeats for each (CK_R1, CK_R2, CK_R3, CK_S1, CK_S2, CK_S3, CK_L1, CK_L2, CK_L3, SS_R1, SS_R2, SS_R3, SS_S1, SS_S2, SS_S3, SS_L1, SS_L2, SS_L3) respectively were collected under the treatment of 300 mmol L^−1^ Na_2_SO_4_ solution at 12 h and the control with water at 12 h. The leaves, stems and roots were used for RNA-seq and real-time fluorescence quantitative PCR (qRT-PCR). All samples were frozen in liquid nitrogen and stored at − 80 °C for further use.

### Measurement of the SOD, POD, Pro and MDA content

The pH of water for plant growth during 0 h, 6 h, 12 h and 24 h was detected by Precision pH meter (S20 Mettler Toledo Instrument Co., LTD). SOD (superoxide dismutase), POD (peroxidase), Pro (proline) and MDA (malondialdehyde) contents in leaves, stems and roots were measured by SOD kits (Nanjing Jiancheng Bioengineering Research Institute), POD kits (Nanjing Jiancheng Bioengineering Research Institute), Pro kits (Nanjing Jiancheng Bioengineering Research Institute) and MDA detection kits (Nanjing Jiancheng Bioengineering Research Institute). The absorbance of SOD, POD, Pro and MDA at 450 nm, 420 nm, 520 nm and 532 nm for 0 h, 6 h, 12 h and 24 h were recorded with three replications for each sample by Ultraviolet–visible spectrophotometer (NanoDrop2000 Seymour Flight).

### RNA extraction, cDNA library construction, and RNA-Seq

Total RNA from each tissue was extracted between control and treatment with 300 mM Na_2_SO_4_, based on the instruction manual of the TRlzol Reagent (Life technologies, California, USA). The integrity and concentration of total RNA was checked by Agilent 2100 Bioanalyzer (Agilent Technologies, Inc., Santa Clara, CA, USA). The isolated mRNA by NEB Next Poly (A) mRNA Magnetic Isolation Module (NEB, E7490) were used for constructing cDNA library through the manufacturer’s instructions of NEBNext Ultra RNA Library Prep Kit for Illumina (NEB, E7530) and NEB Next Multiplex Oligos for Illumina (NEB, E7500). And then, approximately 200nt RNA inserts were used to synthesize the first-strand cDNA and the second cDNA, according to the fragmented mRNA. In the next step, the end-repair/dA-tail and adaptor ligation were performed for double-stranded cDNA, which is to form the cDNA library by Agencourt AMPure XP beads (Beckman Coulter, Inc.) and PCR amplification. Finally, the constructed cDNA libraries of the different samples were sequenced on a flow cell using an Illumina Novaseq 6000 platform. LC-BIO Technologies (http://www.lc-bio.com/about/54.html) provides experimental procedures and commercially performed it.

### Mapping and differential expression genes analysis

Based on the quality results of the paired-end reads, we removed the low-quality reads by per script, which included only adaptor, unknown nucleotides > 5% and Q20 < 20% (percentage of sequences with sequencing error rates < 1%). After filtering from the raw reads, the clean reads were mapped to cotton genome (*G.hirsutum*) by Hisat software^44^. According to the mapped reads from the reference cotton genome, String Tie software^44^ was used to estimate quantification of the gene expression levels with fragments per kilobase of transcript per million fragments mapped (FKPM)^68^. And an edger package, one of R packages, was applied for differential expression analysis between two groups with three tissues respectively. Fold Change ≥ 2 and FDR (false discovery rate) < 0.01 were taken as the threshold of the *P*-value in multiple tests for computing the significance of the differences.

### Gene ontology and pathway enrichment analysis

DEGs for Gene Ontology (GO) terms enrichment analysis implemented by GO seq R packages^69^, were divided into 3 classes, molecular function, cellular process, and biological process. KEGG enrichment analysis of the DEGs was applied to summary the statistical enrichment of differential expression of genes in KEGG pathways (http://www.genome.jp/kegg/) by KOBAS software^70^. The results of the number of genes that mapped to annotated genes in GO and KEGG database was printed using ggplot2 (https://ggplot2.tidyverse.org). The heatmap analysis of DEGs was performed with R (v4.0.2) language software (https://www.r-project.org/)^71–74^ and the model of the regulatory networks in response to Na^+^ stress and SO_4_^2−^ was drawn by Science Slides (http://scienceslides.com/).

### Validation of RNA-Seq by qRT-PCR

Each sample with 3 biological replicates was performed by Real-time RT-PCR (qRT-PCR)^75,76^. A set of 20 genes was chosen randomly by the FPKM. Specific primers for the chosen genes were designed through Primer 3 software. cDNA was synthesized by using an EASY spin Plus Plant RNA Kit (TIANGEN) with gDNA Eraser (TaKaRa)^77^. The qRT-PCR reactions were conducted using a SYBR Green I Master mixture (Bio-Rad, CFX96, Switzerland) according to the manufacturer’s protocol on a Light Cycler 480II system (Bio-Rad, CFX96, Switzerland). The results of qRT-PCR were analysed via the ΔΔCt method^78^, the cotton histone (His) 3 gene (GenBank accession no. AF024716) was used as a standardcontrol^79^. Histone (His) 3 (AF024716) (F: TCAAGACTGATTTGCGTTTCCA, R: GCGCAAAGGTTGGTGTCTTC). Each reaction was carried out in a final volume of 20 µL, 7.8 µL ddH_2_O and containing 10 µL of SYBR Green PCR master mix, 0.4 µL of each gene-specific primer and 1.4 µL of diluted cDNA. The PCR thermal cycling conditions were applied as follows: 95 °C for 5 min; 40 cycles of 95 °C for 5 s, 60 °C for 30 s and 72 °C for 30 s. Data were collected during the extension step: 95 °C for 15 s, 60 °C for 1 min, 95 °C for 30 s and 60 °C for 15 s. Three biological replicates were performed, and three technical replicates were designed per cDNA sample.

## Supplementary Information

Supplementary Information.
