# Supplementary material for: Transcriptome analysis of upland cotton revealed novel pathways to scavenge reactive oxygen species (ROS) responding to Na2SO4 tolerance
Source: Sci Rep. 2021 Apr 21;11:8670. doi: 10.1038/s41598-021-87999-x (PMC8060397; doi:10.1038/s41598-021-87999-x)
Supplement: Supplementary file 1 — Supplementary Information. [file 41598_2021_87999_MOESM1_ESM.doc]

Supplementary Information

**Transcriptome analysis of Upland Cotton revealed novel pathways to scavenge reactive oxygen species (ROS) responding to Na_2_SO_4_ tolerance**

Qinqin Wang, Xuke Lu, Xiugui Chen, Waqar Afzal Malik，Delong Wang, Lanjie Zhao, Junjuan Wang, Shuai Wang, Lixue Guo, Ruifeng Cui, Mingge Han, Cun Rui, Yuexin Zhang, Yapeng Fan, Chao Chen and Wuwei Ye*.

State Key Laboratory of Cotton Biology/ Institute of Cotton Research of Chinese Academy of Agricultural Sciences/ Key Laboratory for Cotton Genetic Improvement, MOA，Anyang, 455000, Henan, China

*Author for correspondence. WY: yew158@163.com

**Supplementary Information contains:**

**
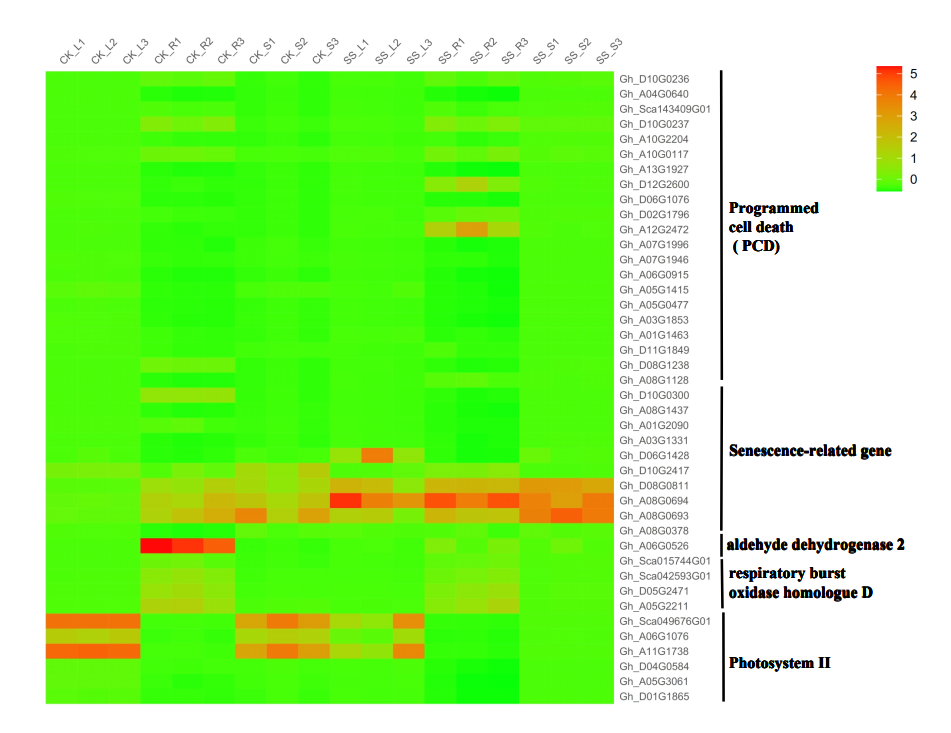
**

**Figure S1.** Heatmap of the standardized FPKM expression level of the DEGs related to programmed cell death and cell senescence among roots, stems and leaves between SS and CK performed by ggplot2 (v3.3.3). CK: Control group; SS: 300 mM Na_2_SO_4_. Red = high expression level of genes, and Green = low expression level of genes.

**
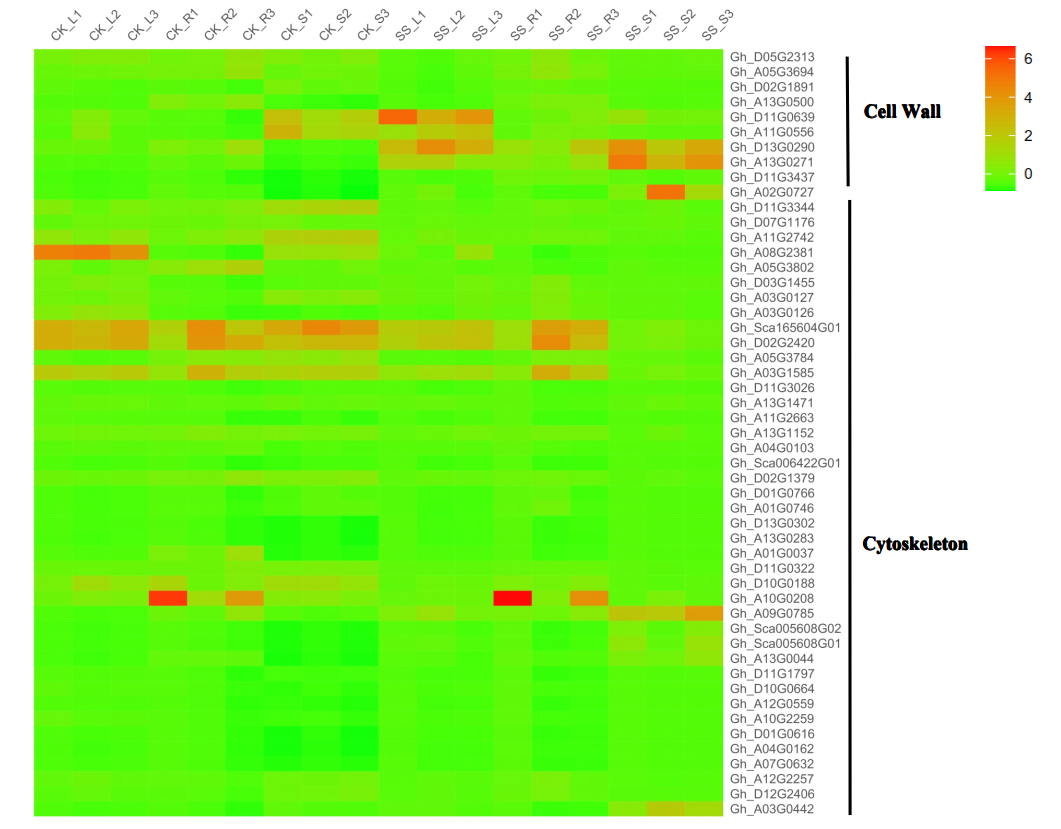
**

**Figure S2.** Heatmap of the standardized FPKM expression level of the DEGs related to cell wall and cytoskeleton among roots, stems and leaves between SS and CK performed by ggplot2 (v3.3.3). CK: Control group; SS: 300 mM Na_2_SO_4_. Red = high expression level of genes, and Green = low expression level of genes.

**
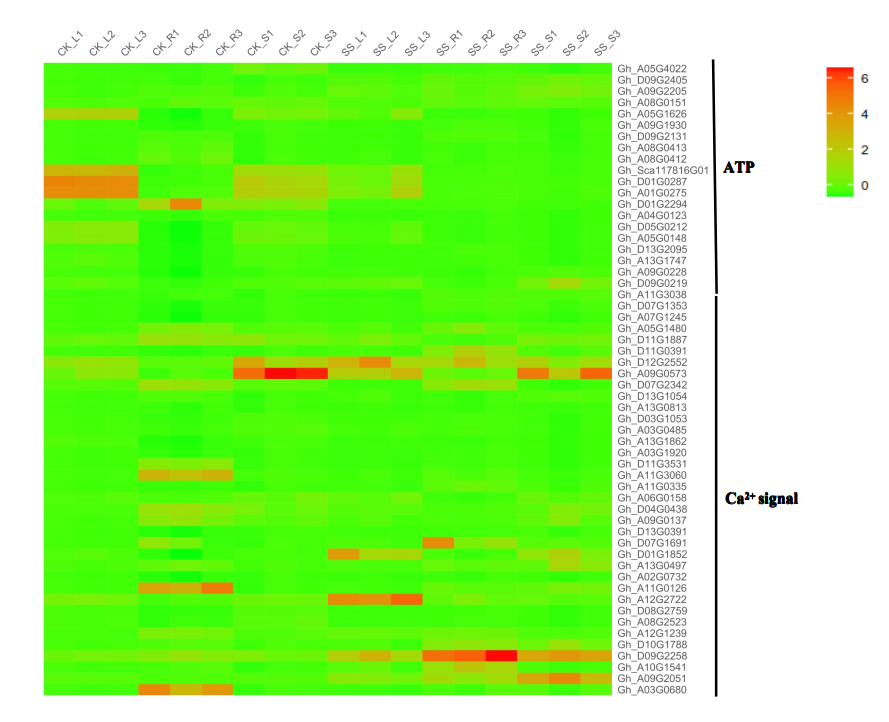
**

**Figure S3.** Heatmap of the standardized FPKM expression level of the DEGs related to Ca^2+^signal and ATP among roots, stems and leaves between SS and CK performed by ggplot2 (v3.3.3). CK: Control group; SS: 300 mM Na_2_SO_4_. Red = high expression level of genes, and Green = low expression level of genes.

**
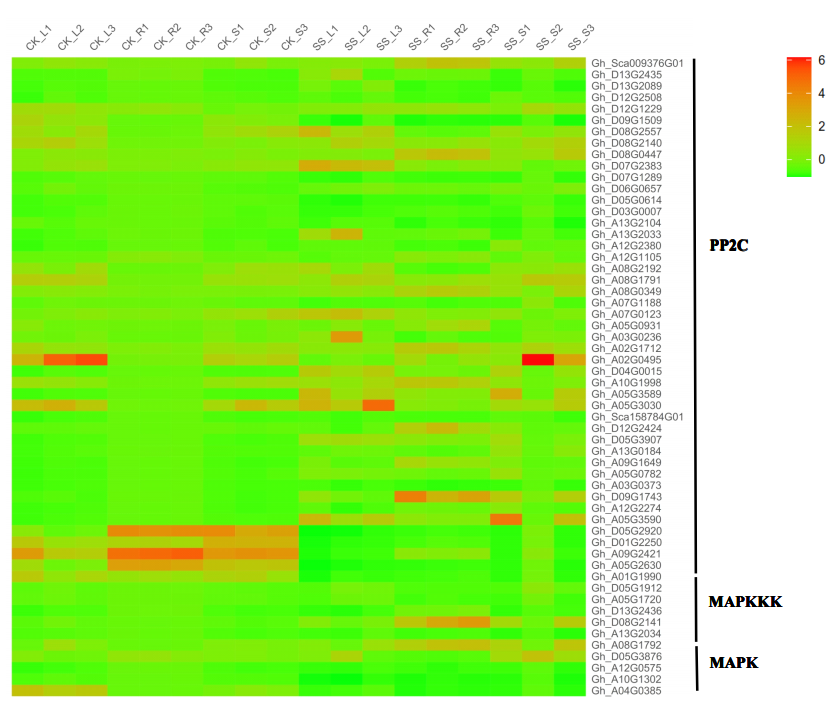
**

**Figure S4.** Heatmap of the standardized FPKM expression level of the DEGs related to PP2C and MAPK among roots, stems and leaves between SS and CK performed by ggplot2 (v3.3.3). CK: Control group; SS: 300 mM Na_2_SO_4_. Red = high expression level of genes, and Green = low expression level of genes.

**
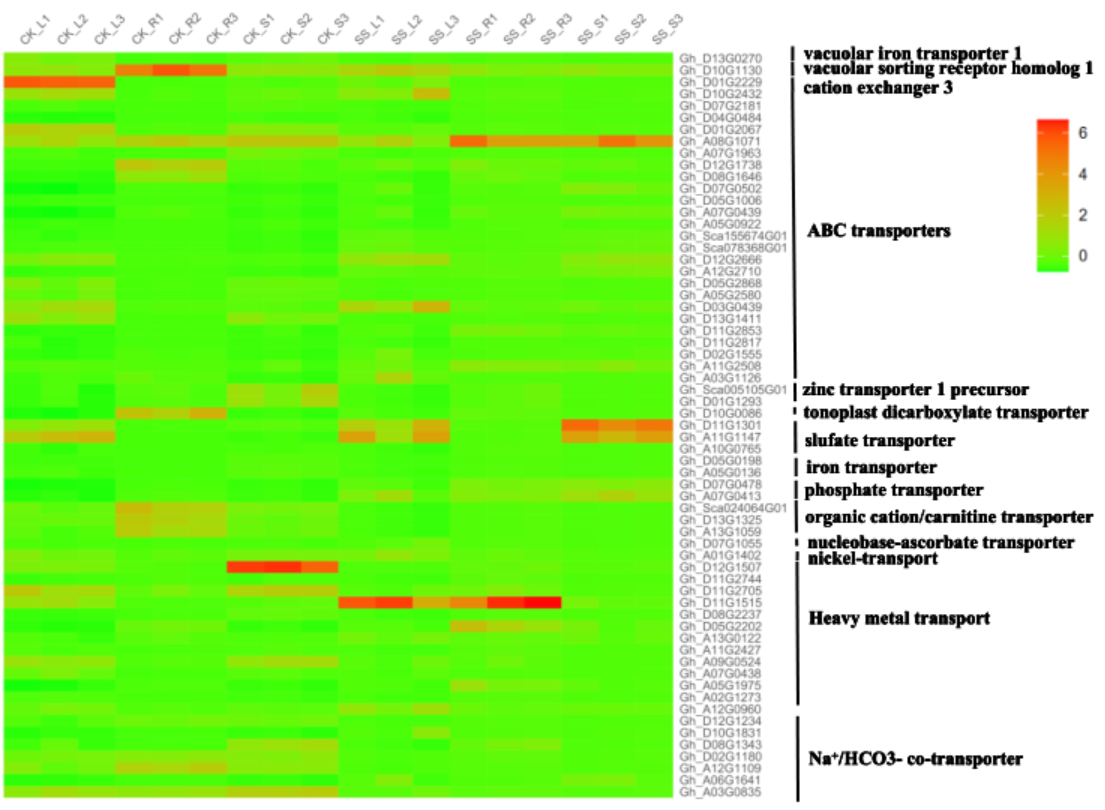
**

**Figure S5.** Heatmap of the standardized FPKM expression level of the DEGs related to ion transport among roots, stems and leaves between SS and CK performed by ggplot2 (v3.3.3). CK: Control group; SS: 300 mM Na_2_SO_4_. Red = high expression level of genes, and Green = low expression level of genes.

**
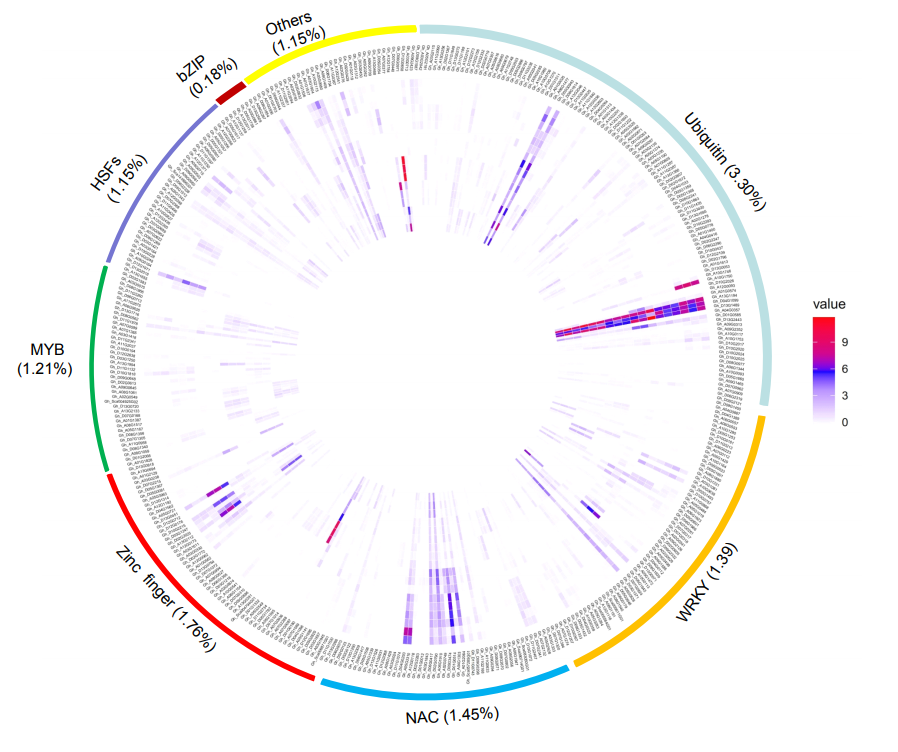
**

**Figure S6.** Heatmap of the standardized FPKM expression level of the DEGs related to multiple transcription factor types (Percentages represent different transcription factors accounted for) among roots, stems and leaves between SS and CK (from the inner to the outer layer: CK_L1, CK_L2, CK_L3, CK_R1, CK_R2, CK_R3, CK_S1, CK_S2, CK_S3, SS_L1, SS_L2, SS_L3, SS_R1, SS_R2, SS_R3, SS_S1, SS_S2, SS_S3) performed by plyr (v1.8.6) and ggplot2 (v3.3.3). CK: Control group; SS: 300 mM Na_2_SO_4_. Red = high expression level of genes, and White = low expression level of genes.

**Table S1. The primer information.**

| Gene ID | Forward primer | Reverse primer |
| --- | --- | --- |
| Gh_A02G1273 | ATCTGGTGGCTCAACCTTACGCTGT | TCACATGACAGAACATGCATTTGGG |
| Gh_A05G1975 | TATGTGCCATACACGCTGGTGGCGA | TTACATGATGGAGCAGGCATTAGGG |
| Gh_A07G0438 | GTGGTGGTGAAGGAGGAGAAGCAAA | TTACATTATAGAACAAGCATTAGGG |
| Gh_A11G2427 | AGAAGTTGAAGATAACCAAGCACCT | TTGATCAGAGTCTCAGCATTTACGT |
| Gh_A12G0960 | AGAAAGAAGATGAAACCAAGAAAGA | TCAACAAATAACACAAGCATTAGGG |
| Gh_A13G0122 | TGAAAGTGGGCTTACATTGTGACGA | TTAATCAGATTCCCAATTGGTTGCT |
| Gh_D04G0145 | ATGTCTCAGACTGTTGTGCTCAAGG | CGGTCTTTGACACGGTCTGCAGCAC |
| Gh_D05G2202 | CCATACAACCTCGTGGCGAACCCTT | TTACATGATGGAGCAGGCATTAGGG |
| Gh_D05G3899 | GATTTACCGACAAAGAAAGTGACAG | TCAGTAATTCATTTTGACCATTGGA |
| Gh_D08G2237 | AAATGCCAATGGTGGGGGTATGTAT | TTACATGATATTGCAGCTGTTTGCA |
| Gh_D11G2705 | ATGAAGTTTCAGACTTGGGTTCTGA | AGCTTCTTAATGAGTATAACCGGAT |
| Gh_D11G2744 | TAACCAAGCACCTCCTCTCAAGTAT | AGTTGGCTTTTACAGTGGCTTTTTG |
| Gh_D12G1507 | ATGGGTGCCTTGGACTCTCTTTCTG | GATTCACCTCCACTGATTTCACACC |
| Gh_A03G0193 | CGGTGCTTTCGCCAAGGTTTATCAT | GACGAATTCCATGACGACATAGATC |
| Gh_A09G1937 | GGGTCGTATGTTGGGACAAGGAAGC | TTGTAGCCATAACTTCGTGGATTTT |
| Gh_D09G1668 | GTGAAGCAAGACTGGCAGTTGGTGA | TTATCGCCTCCTGTTGCCACTGCCT |
| Gh_A13G0863 | ATGGAAGACAAGGATTTAATTGCCA | ATACCCTTCTCCTCCGCATCCGCAA |
| Gh_D10G1130 | AAAGAGAAGAAATATAACAAGGAGT | TGTTTATCACAAGAGTTGGCAGTAT |
| Gh_D05G1637 | ACGATTCCATGCTTTCTCATGCTGC | ATACTCAATCAAAGCAACATCCTCC |
| Gh_A08G0693 | CTAAGCTTTTCTCTACTTTCGTTGT | ATGAAACCTTCTCTTTAGCTCCACC |
